# Enhancing High‐Resolution Assessment in Pain Disorders: Development of an Adaptive Real‐Time Version of the Pain Catastrophizing Scale

**DOI:** 10.1002/ejp.70266

**Published:** 2026-04-20

**Authors:** Maria Roth, Constantin Yves Plessen, Gregor Liegl, Felix Fischer, Lena‐Sophie Koeppen, Peter Vajkoczy, Fabian Prasser, Sandra Nolte, Matthias Rose, Alexander Obbarius

**Affiliations:** ^1^ Department of Psychosomatic Medicine, Center for Internal Medicine and Dermatology, Charité—Universitätsmedizin Berlin Corporate Member of Freie Universität Berlin, Humboldt‐Universität zu Berlin, and Berlin Institute of Health Berlin Germany; ^2^ Psychotherapy and Psychotherapy Research, Center for Psychotherapy University of Graz Graz Austria; ^3^ Center for Patient‐Centered Outcomes Research (CPCOR), Charité—Universitätsmedizin Berlin Corporate Member of Freie Universität Berlin, Humboldt‐Universität zu Berlin, and Berlin Institute of Health Berlin Germany; ^4^ Department of Neurosurgery With Pediatric Neurosurgery Charité—Universitätsmedizin Berlin Berlin Germany; ^5^ Center of Health Data Sciences BIH Berlin Germany; ^6^ Person‐Centred Research, Eastern Health Clinical School Monash University Melbourne Victoria Australia; ^7^ BIH Charite Digital Clinician Scientist Program, Berlin Institute of Health, Charite—Universitatsmedizin Berlin BIH Biomedical Innovation Academy Berlin Germany

**Keywords:** catastrophizing, chronic pain, computer‐adaptive testing, ecological momentary assessment, short‐form construction

## Abstract

**Background:**

Pain Catastrophizing Scale (PCS) scores have consistently been associated with heightened pain perception and poorer treatment outcomes. However, the PCS is typically administered as a trait‐level measure and does not capture within‐day fluctuations in pain‐related cognitions. With increasing interest in real‐time monitoring through Ecological Momentary Assessment (EMA), there is a need for reliable and efficient instruments suitable for repeated use without inducing response fatigue or bias.

**Methods:**

The German version of the 13‐item PCS was adapted using minimal linguistic modifications for momentary assessment. Based on a cross‐sectional design, the original and EMA versions were administered to 691 and 1440 patients, who reported pain within the last week. Using Item Response Theory (IRT), we modeled item characteristics, confirmed unidimensionality, and developed three 4‐item short forms to enable efficient and balanced assessment. In addition, we conducted computer‐adaptive testing (CAT) simulations to examine expected measurement precision and item exposure effects under repeated adaptive use.

**Results:**

The adapted PCS showed strong essential unidimensionality and high item discrimination. All short forms achieved high measurement precision across a broad range of symptom severity. CAT simulations confirmed high precision for initial assessments, though performance declined with repeated use due to the limited item pool.

**Conclusion:**

This study provides an initial psychometric evaluation of a momentary‐adaptive PCS using IRT‐based short forms and CAT simulations. Adaptive administration improved efficiency in early assessments but requires further validation in real‐world EMA designs to establish longitudinal performance and applied utility.

**Significance Statement:**

This study introduces an adaptive, real‐time version of the PCS optimized for ecological momentary assessment. By combining IRT‐based short forms and computer‐adaptive testing, the approach enhances measurement precision while reducing burden in repeated assessments. These findings represent a methodological step toward high‐resolution monitoring of PCS‐based pain‐related cognitions in clinical and mobile health research.

## 1 Introduction

Pain is a major global health issue, causing substantial physical, emotional and socioeconomic burdens (Cohen et al. 2021). One psychological factor influencing pain experience is pain catastrophizing, commonly defined as ‘an exaggerated negative mental set during painful experiences’ (Sullivan et al. 2001). Pain catastrophizing is most commonly operationalized using the Pain Catastrophizing Scale (PCS), a 13‐item self‐report questionnaire assessing rumination, magnification, and helplessness (Sullivan et al. 1995). Higher PCS scores have consistently been associated with poorer pain outcomes, including higher pain intensity (Sullivan et al. 2001) and increased emotional distress (Sullivan et al. 1995; Ziadni et al. 2018), and it has been shown to be relevant across medical and surgical contexts (Gopinath et al. 2019; Sullivan and D'Eon 1990; Sullivan et al. 2005) as well as in the transition from acute to chronic pain (Gopinath et al. 2019).

Despite this strong predictive validity, recent conceptual work has questioned whether instruments such as the PCS fully capture the defining feature of exaggeration implied in the theoretical definition. Many PCS items appear to reflect pain‐related worry, distress or perceived helplessness rather than exaggeration per se (Crombez et al. 2020). Accordingly, PCS scores may more precisely reflect pain‐related negative cognitive‐emotional responses, and the extent to which they represent ‘catastrophizing’ in the strict theoretical sense remains a matter of ongoing discussion.

The PCS is typically administered as a trait‐level measure and is not designed to capture within‐day fluctuations in pain‐related cognitions. Yet evidence suggests that PCS responses are influenced by recent pain experiences and recall processes (Day et al. 2021; Dumenci et al. 2020), indicating both trait‐like and situational components (Campbell et al. 2010; Edwards et al. 2005).

These considerations have motivated efforts to assess pain‐related cognitions with greater temporal resolution. A daily version of the PCS has been introduced (Darnall et al. 2017). However, daily assessments still rely on retrospective aggregation and may obscure short‐lived fluctuations in catastrophizing. To address this limitation, ecological momentary assessment (EMA) designs, involving repeated sampling in naturalistic settings multiple times per day, have increasingly been used to study dynamic pain‐related processes (Frumkin and Rodebaugh 2021; May et al. 2018; Morren et al. 2009). Building on this approach, Frumkin et al. further adapted the PCS for momentary use and increased sensitivity to intraday variation (Frumkin et al. 2023).

Frequent assessment, however, raises psychometric challenges. Individual‐level inference in EMA requires high measurement precision (reliability ≥ 0.9) (Rodebaugh et al. 2016), which static, particularly brief, measures often fail to achieve. Item Response Theory (IRT) can improve precision through adaptive item selection. However, repeated EMA administration introduces additional challenges, including item exposure bias, whereby repeated presentation of identical items may distort responses through fatigue, reduced engagement, or learned expectations (Eisele et al. 2022; Intille et al. 2016; Schneider et al. 2024).

Given these conceptual and methodological considerations, the present study represents an initial step toward developing an adaptive EMA tool for assessing pain‐related cognitions. We aimed to develop a German‐language momentary PCS and to compare its performance with the traditional PCS, using adaptive assessment approaches to improve measurement precision while limiting repeated item exposure.

## 2 Methods

### 2.1 Study Design and Procedure

The current study was based on a cross‐sectional real‐world data collection in in‐ and outpatients of the Department of Psychosomatic Medicine at Charité—Universitätsmedizin Berlin, Germany. Inpatients were assessed in the week after admission, outpatients were assessed at the first visit. Overall, we recruited 2131 patients. Patients answered a set of questionnaires on a tablet computer. The REDcap system (Patridge and Bardyn 2018) was used for data assessment and storage within Charité. The original version of the PCS (*n* = 691; PCS Sample) was assessed between November 2022 and June 2023, while the adapted EMA version of the PCS (*n* = 1440; EMA‐PCS Sample) was assessed between June 2023 and September 2024. Only patients who answered ‘yes’ to the question, ‘Did you have any pain in the last 7 days?’ filled out the questionnaires. Other inclusion criteria were (1) age 18 years or older, (2) ability to read and respond to questionnaire items, (3) sufficient proficiency in the German language, (4) provision of written informed consent. We excluded patients that were not able to answer the psychometric assessment due to a language barrier.

The authors confirm that all procedures related to this work adhere to the ethical standards set by the relevant national and institutional committees on human experimentation, as well as the Helsinki Declaration of 1975, revised in 2008. The study received approval from the Ethics Committees of Charité—Universitätsmedizin Berlin (EA4/297/21), and written informed consent was obtained from all participants following an explanation of the procedure.

### 2.2 Measures

#### 2.2.1 The Pain Catastrophizing Scale (PCS)

We used the German version of the Pain Catastrophizing Scale (Sullivan et al. 1995) to assess pain‐related cognitive‐emotional responses as operationalized by the PCS. The PCS is a 13‐item self‐report questionnaire that assesses three dimensions: rumination, magnification, and helplessness. Each item is scored on a 5‐point response scale from ‘not at all’ to ‘all the time’. The PCS includes questions about the individual's worries regarding whether the pain will end, get worse, how overwhelming or how much the pain hurts among other related worries.

Several studies have investigated the English version of the PCS and found good psychometric properties (Osman et al. 2000, 1997; Van Damme et al. 2002). The psychometric properties of the German version, including internal consistency, construct validity, factor structure and reproducibility, were found to be comparable to those documented in previous studies of the English version (Meyer et al. 2008).

#### 2.2.2 PROMIS Instruments

We utilized the PROMIS‐29 Profile v2.1 (Patient Reported Outcomes Measurement Information System) to assess individuals' quality of life. Developed by the U.S. National Institutes of Health (NIH) in 2004, the PROMIS‐29 Profile is a standardized, generic tool designed to capture patient‐reported outcomes (Cella et al. 2019). The instrument comprises 29 items organized into the following seven distinct domains: pain interference, fatigue, depression, anxiety, sleep disturbance, physical function and ability to participate in social roles and activities, with each of the seven domains consisting of four questions. Additionally, it includes a numerical rating scale (NRS) for assessing pain (Cella et al. 2019). For the purpose of the present study, we specifically evaluated four items related to pain interference and the NRS on pain intensity. In addition to the Profile, we assessed Cognitive Function v2.0 with a 4‐item short form (4a).

### 2.3 Adaptation of PCS to Momentary Version

To adapt the PCS for momentary assessment, several targeted modifications were implemented to support real‐time reporting. The proposed changes were suggested by one individual (A.O.) and reviewed by two others (F.F. and C.Y.P.). The changes to the PCS were discussed and adapted within the research team until a consensus was reached. The adaptation focused on minimal linguistic changes, including the consistent use of present‐tense phrasing and a momentary timeframe, to ensure that items capture current pain‐related cognitions rather than hypothetical or generalized fears.

### 2.4 Statistical Analysis

All statistical analyses were performed using R software (version 4.3.2). The primary objective was to calibrate items from both the conventional static and newly developed EMA version of the PCS onto a unidimensional scale using IRT models. These models were then compared to assess the psychometric performance of each version. Item responses were treated as ordinal data and analysed using graded response models (GRM). For PROMIS instruments, standardized T‐scores were used as continuous variables.

Based on the EMA version of the PCS, we developed short forms that emphasized content balance across PCS subscales while maintaining acceptable measurement precision. Additionally, we simulated CATs to evaluate the efficiency of both the short forms and CAT in accurately capturing the PCS latent trait with fewer items.

#### 2.4.1 Confirmatory Factor Analysis (CFA)

To evaluate the dimensional structure of both the original PCS and the EMA version, CFA was performed using the lavaan package (Rosseel 2012). Two competing models were tested in both the original and EMA version of the PCS. The first was a unidimensional model, which hypothesized that all 13 items of the PCS load onto a single underlying factor. The second model was a three‐factor solution, corresponding to the well‐established subscales of the PCS: Rumination, Magnification and Helplessness (Sullivan et al. 1995). Given the ordinal nature of the data, we used the weighted least squares means and variance adjusted (WLSMV) estimator.

The goodness‐of‐fit of these models was assessed using standard fit indices, including the Comparative Fit Index (CFI), the Tucker–Lewis Index (TLI), the Standardized Root Mean Square Residual (SRMR) and the Root Mean Square Error of Approximation (RMSEA). Fit indices were interpreted according to conventional thresholds, where CFI and TLI values above 0.95, RMSEA values below 0.08, and SRMR below 0.08 were considered indicative of a good fit (Hu and Bentler 1999). These analyses were crucial for determining whether the EMA version of the PCS maintained a similar factor structure to the original PCS, as this would support the validity of the momentary assessment adaptation. In addition, essential unidimensionality is a prerequisite for unidimensional IRT models. If the fit indices did not meet the recommended thresholds, an occurrence not uncommon in the development of item banks (Reeve et al. 2007), essential unidimensionality was further evaluated using bifactor models via the psych package. Two key metrics were used to assess unidimensionality: Explained Common Variance (ECV), with values greater than 0.60 deemed appropriate, and Omega hierarchical (OmegaH), with values above 0.80 indicating sufficient unidimensionality (Rodriguez et al. 2016).

#### 2.4.2 Item Response Theory (IRT) Models

Following the CFA, the psychometric properties of both the static PCS and the EMA version of PCS were examined using Graded Response Models (GRM), implemented via the mirt package (Chalmers 2012). The GRM is particularly well‐suited for ordinal response data, as it models the probability of responding to each item based on the underlying trait and item‐specific parameters (Samejima 1997). For each item in both the original and EMA versions of PCS, we estimated discrimination parameters (a) and thresholds (b1, b2, b3, b4). The discrimination parameter quantifies how well an item distinguishes between individuals with different levels of the latent construct measured by the PCS, while the thresholds indicate the levels of the latent trait required to endorse each response category.

The performance of these models was evaluated using Item Characteristic Curves (ICCs), which visually depict the relationship between the latent trait and the probability of selecting a particular response for each item. Additionally, Test Information Functions (TIFs) were used to assess the precision of each scale across different levels of the PCS latent trait. The TIFs provide a graphical representation of how much information the scale provides at each point on the trait continuum, with higher information reflecting greater measurement precision.

#### 2.4.3 Model Comparison

To directly compare the original PCS with the EMA version of PCS, we visually examined the distribution of latent scores, along with the corresponding ICCs and TIFs for both samples. Also, we evaluated the precision of each model by computing the Root Mean Square Error (RMSE), which quantifies the difference between the observed and predicted responses. Lower RMSE values indicate greater precision.

#### 2.4.4 Convergent Validity

Pearson correlations were calculated between the legacy PCS, the EMA‐adapted PCS, and selected PROMIS domains. Individual‐level IRT model–derived latent trait estimates (*θ* scores) were used for both the legacy PCS and the EMA‐adapted PCS.

#### 2.4.5 Short‐Form Construction

To create efficient short forms of the EMA version of PCS, we employed an iterative item selection process using the TestDesign package in R (Choi et al. 2022). The primary objective was to identify three subsets of 3–4 items that would maximize measurement precision while minimizing the total number of items, making the short forms more practical for frequent, momentary assessments. Items with higher discrimination parameters were prioritized, as they offer greater ability to differentiate between individuals at varying levels of the catastrophizing trait. In addition to discrimination, item thresholds were carefully examined to ensure that the selected items covered a broad range of the catastrophizing continuum. This approach allowed the short forms to remain sensitive to both lower and higher latent PCS levels. To further mitigate item exposure bias, each short form was designed to contain a unique set of items, ensuring minimal overlap.

Once the initial short forms were developed, their performance was assessed by generating TIFs and comparing them to those of the full EMA version of PCS. This comparison allowed us to evaluate whether the short forms maintained sufficient measurement precision across the entire range of the latent PCS continuum. Through multiple iterations of item selection and performance evaluation, the final short forms were optimized to strike a balance between brevity and psychometric robustness.

To verify that the three 4‐item short forms produced interchangeable scores, we performed a one‐way repeated‐measure ANOVA on each participant's T‐scores. Sphericity was assessed via Mauchly's test.

#### 2.4.6 Computer‐Adaptive Testing Simulation

To explore the expected performance of repeated adaptive administrations under controlled conditions, we conducted CAT simulations using model‐generated responses and a fixed, fully calibrated item pool. Specifically, three CAT sessions per participant were simulated using the mirtCAT package in R (Chalmers 2012). Baseline latent trait estimates (thetas) were derived from the IRT model fitted to the cross‐sectional EMA dataset and served as each participant's initial true score.

To simulate temporal dynamics across sessions, we generated theta values for Sessions 2 and 3 by adding Gaussian drift (SD ≈0.40), reflecting session‐to‐session variability consistent with an intraclass correlation of 0.84 as reported by Frumkin et al. (Frumkin et al. 2023). Item responses for Sessions 2 and 3 were then generated using the simdata function from the mirt package based on the perturbed theta values (Chalmers 2012). Perturbed theta values refer to individual latent trait scores that have been adjusted with random variation to simulate changes over time.

We simulated a three‐session CAT, with the additional constraint that previously administered items were excluded in subsequent sessions to mimic real‐world adaptive assessment. Item selection was based on the Maximum Posterior Weighted Information (MPWI) criterion, and theta was estimated using the Maximum A Posteriori (MAP) method. For each session, the theta estimate from the prior session was used as the starting value. To facilitate comparability with fixed short forms, the length of each CAT session was constrained to four items per session. Content balancing was implemented using content probabilities in mirtCAT. In particular, the probability to select items from one of the three PCS domains Rumination, Magnification, and Helplessness was equal (i.e., 0.333). For each session, the estimated theta, standard error, and item set were recorded.

## 3 Results

### 3.1 Sample Characteristics

Sample characteristics are summarized in Table 1. Participants in both groups (PCS, EMA‐PCS) were on average 43 years old, mostly female (PCS = 70%, EMA‐PCS = 64%), predominantly had a high school degree (PCS = 59%, EMA‐PCS = 56%), were employed (PCS = 61%, EMA‐PCS = 58%), and more than half were in a relationship (PCS = 54%, EMA‐PCS = 53%).

**TABLE 1:** Sociodemographic characteristics.

	PCS (*N* = 691)	EMA‐PCS (*N* = 1440)
Age in years, M ± SD (range)	43.2 ± 14.7 (18–87)	43 ± 15.3 (18–87)
Sex (%)		
Female	70	64.3
Male	28.7	34.8
Diverse	1.3	1
Relationship status (%)		
Partner	54.3	53.9
No partner	45.7	46.2
Children (%)		
Children	44.4	42.2
No children	55.6	57.8
Educational level (%)		
Degree of primary education	5.7	6.9
Degree of secondary education	32.5	34.2
High school	59.3	56.6
No graduation	2.6	2.3
Professional qualification, *n* (%)		
Vocational training	35.9	36.2
University degree	42.6	38.4
No professional qualification	15.3	18.2
Other	6.2	7.2
Work status, *n* (%)		
Full‐time	36.2	32.5
Part‐time	18.7	17.3
Seeking employment	6.3	8.5
Not working	30.2	30.3
Other	8.6	11.5
Self‐reported health in the past 7 days, M ± SD in T‐score		
Physical function	42.6 ± 7.9	42.4 ± 8.0
Sleep disturbance	56.8 ± 8.4	57.1 ± 8.2
Fatigue	60.8 ± 9.0	61.3 ± 8.9
Pain interference	61.5 ± 7.5	61.4 ± 7.8
Anxiety	60.0 ± 9.5	61.1 ± 9.3
Depression	60.4 ± 9.2	61.1 ± 8.9
Social participation	41.4 ± 8.5	41.3 ± 8.3
Cognitive function	45.4 ± 11.0	45.2 ± 11.1

*Note:* 9%–11% missing values in the variables were excluded in the PCS group. 6%–8% missing values in the variables were excluded in the EMA‐PCS group. Percentages calculated in the table are based on the valid cases for each variable and group.

### 3.2 Adaptation of PCS to EMA

To adapt the PCS for momentary assessment, the item instructions and wording were modified to emphasize current pain‐related experiences. Specifically, the item instructions prompt test‐takers to report on their thoughts and feelings ‘in the moment’ when they are in pain, encouraging responses based on immediate pain experiences rather than generalized or hypothetical concerns. In addition, item wording was adjusted to reflect present‐tense phrasing; for example Item 1 was modified from ‘I worry all the time about whether the pain will end’ to ‘I worry about whether the pain will ever end’, and Item 6 from ‘I become afraid that the pain will get worse’ to ‘I am afraid that the pain will get worse’.

The response scale was also adapted to align with momentary reporting, ranging from ‘Not at all’ to ‘Completely’ instead of the original ‘Not at all’ to ‘All the time’.

For the complete version of the momentary PCS in English and German, please refer to Tables S1 and S2.

### 3.3 IRT Model Estimation and Comparison

The fit indices for the legacy (static) and EMA versions of the PCS under different factor models are presented in Table 2. In the legacy sample, the one‐factor model showed poor fit, with a CFI of 0.846, TLI of 0.815, and RMSEA of 0.179. The three‐factor model, reflecting the subdomains, significantly improved the fit (CFI = 0.934, TLI = 0.917, RMSEA = 0.120). The bifactor model yielded the best fit (CFI = 0.967, TLI = 0.953, RMSEA = 0.091) and demonstrated strong essential unidimensionality (ECV = 0.86, Omega = 0.96, OmegaH = 0.92). Comparable results were found in the EMA sample, where the one‐factor solution had a CFI of 0.831, TLI of 0.797, and RMSEA of 0.158. The three‐factor model improved these fit indices (CFI = 0.906, TLI = 0.882, RMSEA = 0.137), and again, the bifactor model provided the best fit (CFI = 0.961, TLI = 0.945, RMSEA = 0.093).

**TABLE 2:** Fit indices for static PCS and PCS‐EMA models.

Model	Sample	*N*	df	*χ* ^2^	CFI	TLI	RMSEA (90% CI)	SRMR	ECV	Omega	OmegaH
One factor	PCS	691	65	1098.35	0.846	0.815	0.179 (−)	0.055	—	—	—
Three factor	PCS	691	62	549.20	0.934	0.917	0.120 (0.111–0.130)	0.036	—	—	—
Bifactor	PCS	691	54	246.28	0.967	0.953	0.091 (0.080–0.101)	0.022	0.86	0.96	0.92
One factor	EMA‐PCS	1440	65	2390.76	0.831	0.797	0.158 (−)	0.060	—	—	—
Three factor	EMA‐PCS	1440	62	1403.68	0.906	0.882	0.137 (0.130–0.143)	0.043	—	—	—
Bifactor	EMA‐PCS	1440	55	523.37	0.961	0.945	0.093 (0.086–0.100)	0.027	0.83	0.96	0.92

Abbreviations: χ^2^, Chi‐squared; CFI, comparative fit index; CI, confidence interval; df, degrees of freedom; ECV, explained common variance; EMA, Ecological Momentary Assessment; *N*, number of patients; OmegaH, Omega Hierarchical; PCS, Pain Catastrophizing Scale; RMSEA, root mean square error of approximation; SRMR, standardized root mean square residual; TLI, Tucker–Lewis Index.

Item parameter estimates for the PCS‐EMA scale, based on a graded response model, are shown in Table 3. Across items, discrimination parameters ranged from 1.27 to 3.95, indicating that the items are highly sensitive to differences in levels of the latent PCS trait. The threshold parameters were well distributed along the latent continuum, and item‐level fit statistics (*p*(*S* − *χ* ^2^)) were generally acceptable. These results support the unidimensional calibration of the EMA version of the PCS.

**TABLE 3:** Item content and IRT parameter estimates for the EMA version of PCS.

Item	Item content	*p*(*S* − *χ* ^2^)	a	b1	b2	b3	b4
pc1m	I worry whether the pain will end	0.46	2.29	−1.46	−0.85	−0.13	0.60
pc2m	I feel I can't go on	0.05	2.90	−0.95	−0.28	0.53	1.32
pc3m	It's terrible and I think it's never going to get any better	0.76	3.18	−0.85	−0.13	0.60	1.40
pc4m	It's awful and I feel that it overwhelms me	0.23	3.95	−0.56	0.10	0.76	1.43
pc5m	I feel I can't stand it anymore	0.06	3.44	−0.68	−0.02	0.68	1.43
pc6m	I am afraid that the pain will get worse	0.50	2.29	−1.30	−0.60	0.18	1.14
pc7m	I think of other painful events	0.68	1.57	−0.69	0.22	1.13	2.02
pc8m	I anxiously want the pain to go away	0.92	2.90	−1.24	−0.68	−0.11	0.64
pc9m	I can't seem to keep it out of my mind	0.11	2.93	−0.74	0.00	0.79	1.55
pc10m	I think about how much it hurts	0.03	2.64	−0.77	−0.00	0.85	1.68
pc11m	I think about how badly I want the pain to stop	0.24	2.57	−1.22	−0.67	0.01	0.82
pc12m	There's nothing I can do to reduce the intensity of the pain	0.08	1.91	−1.02	−0.14	0.82	1.76
pc13m	I wonder whether something serious may happen	0.25	1.27	−1.15	−0.17	0.79	1.80

*Note:* *p*(*S* − *χ* ^2^) = item‐level *S* − *χ* ^2^ fit statistic (*p*‐value). a = item discrimination parameter. b1–b4 = threshold parameters, pc1m = momentary version of the first item of pain catastrophizing scale.

### 3.4 Associations With PROMIS Health Domains

Both PCS versions showed moderate to strong positive correlations with PROMIS Pain Interference, Depression, Anxiety, and Fatigue, and moderate negative correlations with Physical Function, Cognitive Function, and Ability to Participate in Social Roles and Activities (Table 4). The magnitude and pattern of correlations were highly similar for PCS and PCS‐EMA, supporting convergent validity and conceptual continuity of PCS scores in their momentary‐adapted form.

**TABLE 4:** Correlations between PCS, PCS‐EMA and PROMIS domains.

PROMIS domain	*r*	*r*
	PCS	PCS_EMA
Physical function	−0.29	−0.27
Sleep disturbance	0.37	0.30
Fatigue	0.44	0.39
Pain interference	0.58	0.58
Anxiety	0.57	0.50
Depression	0.58	0.51
Ability to participate in social roles and activities	−0.47	−0.45
Cognitive function	−0.36	−0.36

*Note:* All correlations *p* < 0.001.

Abbreviations: PCS, Pain Catastrophizing Scale; PCS_EMA, momentary version of the PCS; *r*, Pearson correlation coefficient.

### 3.5 Creation of PCS Short Forms

In order to reduce respondent burden while preserving measurement precision, we derived several brief, parallel short forms from the full EMA‐adapted PCS item bank using an iterative item selection procedure within the TestDesign framework. The item selection was guided by constraints that ensured each short form included at least one item from each PCS subdomain (Helplessness, Magnification, and Rumination) and that the total number of items was fixed (in this instance, 4 items per short form). After several iterations, the optimal 4‐item short forms were identified. Table 5 summarizes the final short forms, listing for each form the item code, the corresponding subdomain, and the adapted item content.

**TABLE 5:** Final short forms derived from the PCS‐EMA.

Short form	Item code	Domain	Adapted item content
SF‐1	pc1m	Helplessness	I worry whether the pain will ever end.
SF‐1	pc2m	Helplessness	I feel I can't go on.
SF‐1	pc7m	Magnification	I think of other painful events.
SF‐1	pc9m	Rumination	I can't seem to keep it out of my mind.
SF‐2	pc3m	Helplessness	It's terrible and I think it's never going to get any better.
SF‐2	pc8m	Rumination	I anxiously want the pain to go away.
SF‐2	pc10m	Rumination	I think about how much it hurts.
SF‐2	pc13m	Magnification	I wonder whether something serious may happen.
SF‐3	pc5m	Helplessness	I feel I can't stand it anymore.
SF‐3	pc6m	Magnification	I am afraid that the pain will get worse.
SF‐3	pc11m	Rumination	I think about how badly I want the pain to stop.
SF‐3	pc12m	Helplessness	There's nothing I can do to reduce the intensity of the pain.

Abbreviations: pc1m, momentary version of the first item of pain catastrophizing scale; SF1, short form 1.

**FIGURE 1:**
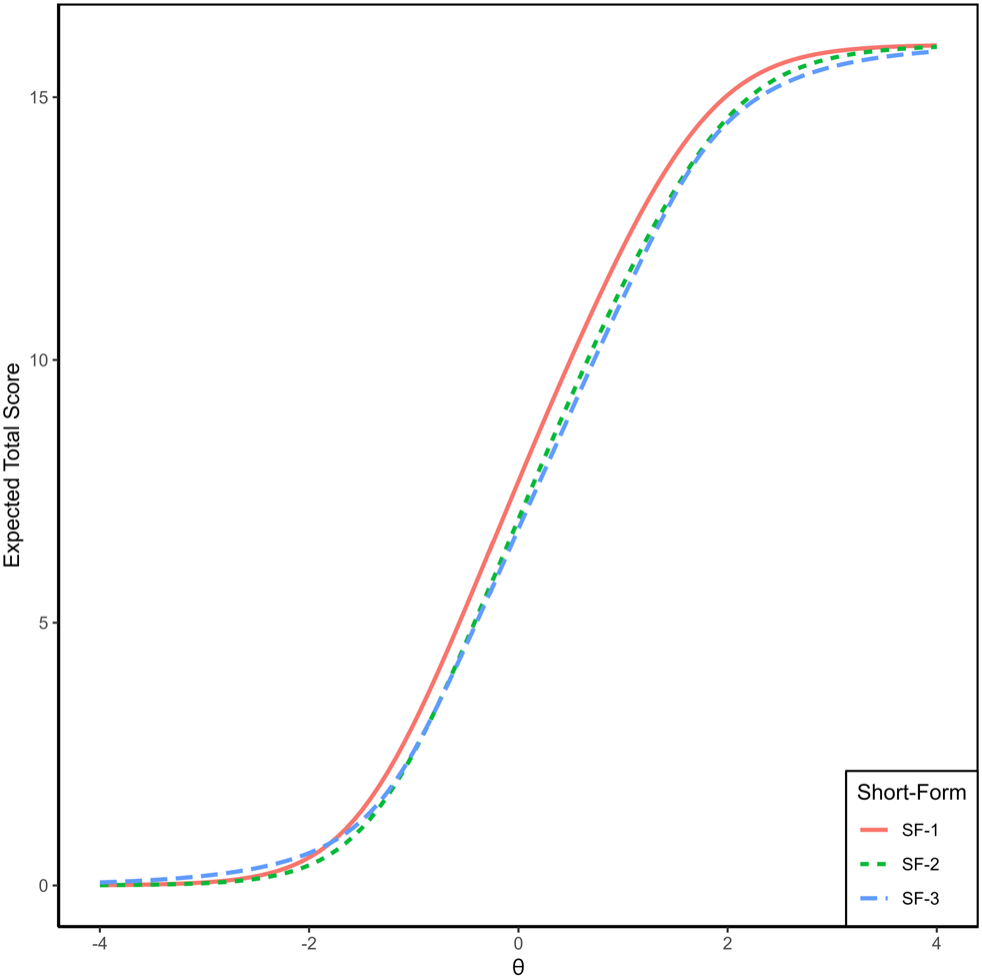
Expected total scores across the latent trait continuum (theta) for three short forms of the EMA‐adapted Pain Catastrophizing Scale. The curves depict the model‐based expected raw scores for each short form as a function of theta.

In addition to the summary table, we evaluated the measurement properties of the short forms by comparing the model‐based expected total scores across the latent trait continuum (theta). Figure 1 displays the expected raw scores for each short form as a function of theta. The highly similar curves across all three forms indicate that they produce comparable expected scores over a broad range of the latent trait.

A repeated‐measures ANOVA showed no significant differences in T‐scores across the three 4‐item short forms (Greenhouse–Geisser–corrected *F*(1.97, 2832.67) = 0.0007, *p* = 0.999), indicating that the three short forms yield statistically equivalent scores.

### 3.6 CAT Simulation

**FIGURE 2:**
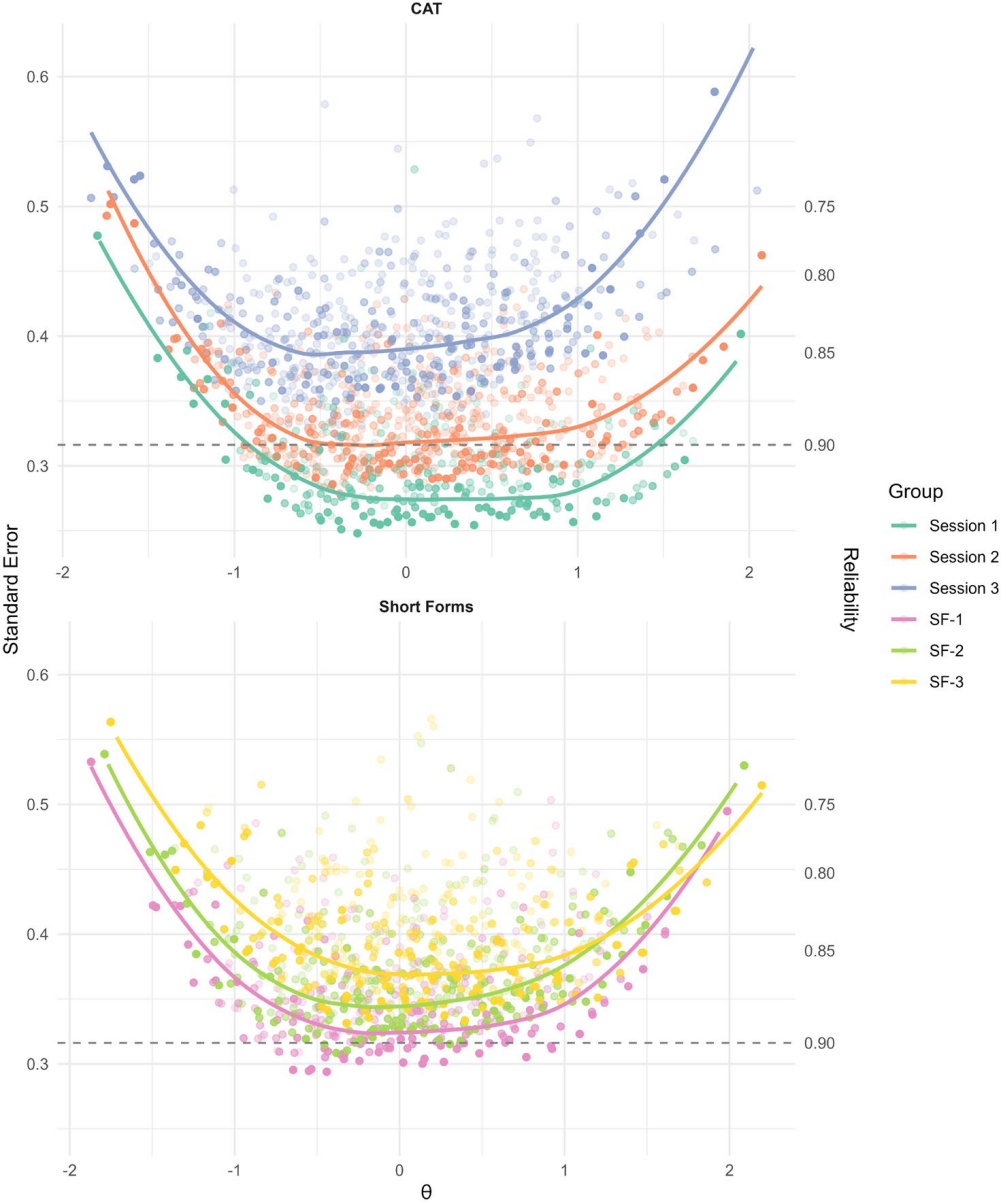
Measurement precision of short forms versus CAT.

The CAT simulations across three sessions demonstrated high measurement precision in the first administration, with standard errors consistently below 0.35 and marginal reliability exceeding 0.90 in the theta range between −1 and +1.5. CATs in Sessions 2 and 3 showed reduced precision: Session 2 maintained marginal reliability > 0.90 between approximately −0.75 and +0.5, whereas Session 3 fell below the 0.90 threshold across the full theta range. Compared to the static short forms, CATs in Sessions 1 and 2 showed higher measurement precision, whereas in Session 3 their precision was lower (Figure 2).

The adaptive algorithm effectively minimized item overlaps across sessions while maintaining robust estimation of theta (*θ*) scores on the latent trait. Notably, in the first CAT session, that is when the algorithm was able to select from the whole pool of items, Item 4 (‘Right now, it feels awful and like it could overwhelm me’; Helplessness dimension) was selected as the starting item in 100%, and Item 6 (‘Right now, I'm afraid the pain will get worse’, Magnification) was selected as the second item in 100%. This was followed by either Item 8 (55.4% of the cases, Rumination) or 9 (44.6%, Rumination) as third item, followed by 1 (7.9%, Helplessness), 3 (7.6%, Helplessness), or 5 (85.0%, Helplessness) as fourth item. The average administration order for these items confirms their early selection, reflecting their high information contribution early in the adaptive assessment process.

## 4 Discussion

Given the increasing interest in accurate real‐time assessment of chronic pain (May et al. 2018; Morren et al. 2009), the present study developed and evaluated a German‐language adaptive momentary Pain Catastrophizing Scale, demonstrating strong psychometric properties and improved measurement efficiency through short forms and CAT‐based approaches. Moreover, we sought to minimize item exposure bias and achieve a balanced content representation by implementing adaptive short forms and CAT algorithms, thereby improving the efficiency and accuracy of the assessments. Supporting construct validity, both the legacy PCS and the EMA‐adapted PCS showed highly similar associations with relevant PROMIS domains, including pain interference, emotional distress, fatigue and functioning. Together, these findings indicate improvements in the efficiency and precision of PCS‐based assessment of pain‐related cognitions while preserving continuity with the established PCS construct.

The EMA version of the German PCS required only minor adjustments to the original German version, which consists of 13 items. Both the PCS and EMA‐PCS samples demonstrated strong essential unidimensionality in the bifactor model as a part of IRT analyses. Furthermore, the item parameter estimates support the unidimensionality of the EMA version of the PCS. To improve measurement efficiency, we created three different 4‐item short forms that included at least one item from each subdomain of the PCS, that is Helplessness, Magnification, and Rumination. This approach minimizes response time while maintaining measurement accuracy. Our results indicate that each short form reliably estimates the PCS latent trait with acceptable measurement precision.

In addition to static short forms, we conducted simulated CAT sessions to examine the expected performance of repeated adaptive administrations under controlled conditions. The first CAT session showed high measurement precision, with marginal reliability exceeding 0.90 across a broad range of the latent trait (approximately *θ* = −1 to +1.5). Measurement precision declined in subsequent sessions as the available item pool was reduced by item exclusion constraints: in Session 2, reliability remained above 0.90 only within a narrower theta range (approximately *θ* = −0.75 to +0.5), whereas Session 3 fell below conventional reliability thresholds across the full continuum. Relative to the static short forms, CATs provided higher precision in Sessions 1 and 2, but lower precision in Session 3. These findings reflect limitations imposed by item pool size when CATs are applied repeatedly within short timeframes. Importantly, the CAT results are based on simulations using model‐generated responses and a fixed, fully calibrated item pool, and therefore represent an idealized evaluation of adaptive measurement performance (Chalmers 2012). Although conservative constraints were applied (item exclusion across sessions and simulated trait drift), the resulting reliability estimates should be interpreted as theoretical performance limits rather than empirical evidence from real‐world EMA applications. Future studies implementing the momentary‐adaptive PCS in actual EMA designs are needed to evaluate its longitudinal performance under applied conditions.

An analysis of item usage showed that certain items, particularly from the Rumination subscale, were frequently selected as initial items across simulated CAT sessions. The most commonly selected starting items included ‘I can't seem to keep it out of my mind’ (Item 9), ‘I keep thinking about how much it hurts’ (Item 10), and ‘I anxiously want the pain to go away’ (Item 8). These items showed high discrimination parameters and contributed substantial information early in the adaptive assessment process. Their frequent early selection reflects their information value within the calibrated item pool and may inform future investigations of item prioritization in CAT algorithms without implying differential conceptual importance of specific subdomains.

The findings further illustrate practical trade‐offs involved in applying CATs to momentary assessment contexts. Adaptive testing can offer efficiency and high precision when a sufficiently large item pool is available (Gibbons 2017; Rose et al. 2012); however, repeated administrations within short timeframes may lead to reduced precision as item availability becomes constrained (Schneider et al. 2024). Several strategies may therefore be considered in future applications, including limiting the frequency of CAT administrations within short intervals, expanding the item pool to support repeated assessments, or allowing controlled item overlap across sessions for highly informative items. The suitability of these approaches will depend on the intended application, such as clinical monitoring, observational EMA studies, or adaptive digital health interventions.

Previous work has demonstrated both the promise and limitations of brief adaptations of the PCS for intensive assessment contexts. Darnall et al. developed a brief 3‐item daily PCS version (Darnall et al. 2017) that substantially reduced respondent burden but may be susceptible to item exposure effects due to repeated presentation of a small fixed item set (Intille et al. 2016; Schneider et al. 2024). Frumkin et al. adapted the English PCS for momentary assessment by modifying item wording to capture immediate pain‐related experiences (Frumkin et al. 2023). In their study, momentary PCS scores were associated with postoperative pain trajectories and daily fluctuations in pain during the first month after surgery, supporting the potential added value of momentary assessment relative to single retrospective trait measures.

Building on this work, the present study introduced a German‐language adaptation of the PCS for momentary use and evaluated its psychometric properties using IRT‐based short forms and CAT simulations. While the results support the feasibility of adaptive momentary assessment of the PCS latent trait, the utility in applied research and clinical practice remains to be established. In particular, future studies should examine its predictive validity, sensitivity to change, and feasibility in diverse patient populations and clinical contexts, including perioperative care, chronic pain management, and psychosomatic treatment settings.

Growing interest in Just‐in‐Time Adaptive Interventions (JITAIs) underscores the need for precise and low‐burden momentary assessment tools, as such interventions rely on accurate real‐time data to inform decision rules and tailoring variables (Collins et al. 2004; Nahum‐Shani et al. 2015, 2018). The German adaptive momentary PCS may represent a candidate measure for this purpose, provided that its performance in real‐world EMA designs is established. Recent work on psychometrically optimized adaptive EMA frameworks suggests that adaptive assessment strategies can improve efficiency and temporal precision in the measurement of dynamic psychological states (Schneider et al. 2024). Based on previous EMA research, a sampling frequency of approximately three to five momentary assessments per day appears suitable for capturing fluctuations in situational pain‐related cognitions and supporting adaptive decision‐making (Frumkin et al. 2023). Within such frameworks, elevated momentary PCS scores could be used to trigger brief, low‐burden intervention components, such as relaxation‐based exercises or activity‐prompting strategies, which have shown feasibility in mobile health contexts (Carroll and Seers 1998; Sitthipornvorakul et al. 2018).

## 5 Strengths and Limitations

This study has several strengths. To our knowledge, it is the first to develop and evaluate an EMA‐adapted German version of the PCS, including IRT‐based short forms and CAT approaches. Large, well‐characterized samples were used to calibrate the item parameters and validate the adaptive instruments, increasing the robustness and clinical relevance of the findings. The combined use of bifactor modeling, IRT calibration, short‐form construction, and CAT simulations provides a comprehensive framework for evaluating structural validity and adaptive precision.

Several limitations should be acknowledged. Most importantly, the study relied on cross‐sectional data. Although repeated CAT administrations were simulated to approximate longitudinal use, no empirical EMA or CAT data across time points were collected. Accordingly, the CAT simulations represent an idealized evaluation of adaptive measurement performance under controlled conditions and are likely to yield upper‐bound estimates of precision relative to real‐world EMA applications. As a result, the ability of the momentary‐adaptive PCS to capture within‐person change over time remains to be established.

Although alternative EMA strategies such as coverage‐based EMA have been proposed to capture infrequent or short‐lived experiences (Shiffman et al. 2008; Stone et al. 2023), their applicability to pain‐related cognitions remains largely untested; accordingly, the present work focused on strictly momentary assessment as a first step in instrument development.

Patient partners were not involved in reviewing the items or testing the EMA version, which limits insight into item relevance, comprehensibility and ecological validity. This was a deliberate decision at this early stage of instrument development, as item modifications were intentionally kept minimal to preserve the original item content and construct coverage. Incorporating patient perspectives and evaluating the instrument in real‐world EMA designs represent important next steps for future research.

In addition, recent conceptual work has questioned whether commonly used pain catastrophizing instruments, including the PCS, fully capture catastrophizing as theoretically defined, noting overlap with related constructs such as pain‐related worry or distress (Crombez et al. 2020, 2023). While the PCS remains a robust predictor of pain‐related outcomes, scores should therefore be interpreted with caution in light of potential conceptual overlap.

Generalizability is further limited by the psychosomatic outpatient sample. Information on pain chronicity, location and momentary pain status was not available, and the inclusion criterion of pain within the past 7 days likely resulted in a heterogeneous mix of acute and chronic pain presentations.

Finally, CAT performance declined across simulated sessions due to item pool constraints and item reuse restrictions. While CATs demonstrated higher precision than static short forms in early administrations, later sessions fell below recommended reliability thresholds, underscoring the importance of sufficiently large item pools for sustained adaptive assessment.

## 6 Conclusion

This study presents an initial psychometric evaluation of a momentary‐adaptive PCS using IRT‐based short forms and CAT simulations. While adaptive assessment improved efficiency in early administrations, sustained precision was limited by item pool size. Future work should expand the item bank and evaluate the instrument in real‐world EMA designs to establish longitudinal performance and applied utility.

## Author Contributions

This study was designed by Alexander Obbarius., Matthias Rose, Peter Vajkoczy and Fabian Prasser. Alexander Obbarius, Matthias Rose, and Sandra Nolte acquired funding for the study. The data were analysed by Constantin Yves Plessen, Alexander Obbarius, Lena‐Sophie Koeppen, and Maria Roth, and the results were critically examined by all authors. Maria Roth had a primary role in preparing the manuscript, which was edited by Alexander Obbarius. All authors have approved the final version of the manuscript and agree to be accountable for all aspects of the work.

## Funding

Dr. Obbarius is a participant in the BIH Charité Digital Clinician Scientist Program funded by Charité—Universitätsmedizin Berlin and the Berlin Institute of Health at Charité (BIH).

## Conflicts of Interest

The authors declare no conflicts of interest.

## Supporting information


**Table S1:** German changes and version of the momentary PCS.

**Table S2:** English changes and version of the momentary PCS.

## References

[ejp70266-bib-0001] Campbell, C. M. , T. Kronfli , L. F. Buenaver , et al. 2010. “Situational Versus Dispositional Measurement of Catastrophizing: Associations With Pain Responses in Multiple Samples.” Journal of Pain 11, no. 5: 443–453.e442. 10.1016/j.jpain.2009.08.009. 20439057 PMC2898132

[ejp70266-bib-0002] Carroll, D. , and K. Seers . 1998. “Relaxation for the Relief of Chronic Pain: A Systematic Review.” Journal of Advanced Nursing 27, no. 3: 476–487. 10.1046/j.1365-2648.1998.00551.x. 9543032

[ejp70266-bib-0003] Cella, D. , S. W. Choi , D. M. Condon , et al. 2019. “PROMIS() Adult Health Profiles: Efficient Short‐Form Measures of Seven Health Domains.” Value in Health 22, no. 5: 537–544. 10.1016/j.jval.2019.02.004. 31104731 PMC7201383

[ejp70266-bib-0004] Chalmers, R. P. 2012. “Mirt: A Multidimensional Item Response Theory Package for the R Environment.” Journal of Statistical Software 48, no. 6: 1–29. 10.18637/jss.v048.i06.

[ejp70266-bib-0005] Choi, S. W. , S. Lim , and W. J. van der Linden . 2022. “TestDesign: An Optimal Test Design Approach to Constructing Fixed and Adaptive Tests in R.” Behaviormetrika 49: 191–229. 10.1007/s41237-021-00145-9.

[ejp70266-bib-0006] Cohen, S. P. , L. Vase , and W. M. Hooten . 2021. “Chronic Pain: An Update on Burden, Best Practices, and New Advances.” Lancet 397, no. 10289: 2082–2097. 10.1016/S0140-6736(21)00393-7. 34062143

[ejp70266-bib-0007] Collins, L. M. , S. A. Murphy , and K. L. Bierman . 2004. “A Conceptual Framework for Adaptive Preventive Interventions.” Prevention Science 5, no. 3: 185–196. 10.1023/b:prev.0000037641.26017.00. 15470938 PMC3544191

[ejp70266-bib-0008] Crombez, G. , A. L. De Paepe , E. Veirman , C. Eccleston , G. Verleysen , and D. M. L. Van Ryckeghem . 2020. “Let's Talk About Pain Catastrophizing Measures: An Item Content Analysis.” PeerJ 8: e8643. 10.7717/peerj.8643. 32181053 PMC7060750

[ejp70266-bib-0009] Crombez, G. , E. Veirman , D. Van Ryckeghem , W. Scott , and A. De Paepe . 2023. “The Effect of Psychological Factors on Pain Outcomes: Lessons Learned for the Next Generation of Research.” Pain Reports 8, no. 6: e1112. 10.1097/pr9.0000000000001112. 38027466 PMC10631620

[ejp70266-bib-0010] Darnall, B. D. , J. A. Sturgeon , K. F. Cook , et al. 2017. “Development and Validation of a Daily Pain Catastrophizing Scale.” Journal of Pain 18, no. 9: 1139–1149. 10.1016/j.jpain.2017.05.003. 28528981 PMC5581222

[ejp70266-bib-0011] Day, M. A. , G. Young , and M. P. Jensen . 2021. “Differentiating State Versus Trait Pain Catastrophizing.” Rehabilitation Psychology 66, no. 1: 39–49. 10.1037/rep0000318. 32212754

[ejp70266-bib-0012] Dumenci, L. , K. Kroenke , F. J. Keefe , et al. 2020. “Disentangling Trait Versus State Characteristics of the Pain Catastrophizing Scale and the PHQ‐8 Depression Scale.” European Journal of Pain 24, no. 8: 1624–1634. 10.1002/ejp.1619. 32538517 PMC7686072

[ejp70266-bib-0013] Edwards, R. R. , C. M. Campbell , and R. B. Fillingim . 2005. “Catastrophizing and Experimental Pain Sensitivity: Only In Vivo Reports of Catastrophic Cognitions Correlate With Pain Responses.” Journal of Pain 6, no. 5: 338–339. 10.1016/j.jpain.2005.02.013. 15890636

[ejp70266-bib-0014] Eisele, G. , H. Vachon , G. Lafit , et al. 2022. “The Effects of Sampling Frequency and Questionnaire Length on Perceived Burden, Compliance, and Careless Responding in Experience Sampling Data in a Student Population.” Assessment 29, no. 2: 136–151. 10.1177/1073191120957102. 32909448

[ejp70266-bib-0015] Frumkin, M. R. , J. K. Greenberg , P. Boyd , et al. 2023. “Establishing the Reliability, Validity, and Prognostic Utility of the Momentary Pain Catastrophizing Scale for Use in Ecological Momentary Assessment Research.” Journal of Pain 24, no. 8: 1423–1433. 10.1016/j.jpain.2023.03.010. 37019164

[ejp70266-bib-0016] Frumkin, M. R. , and T. L. Rodebaugh . 2021. “The Role of Affect in Chronic Pain: A Systematic Review of Within‐Person Symptom Dynamics.” Journal of Psychosomatic Research 147: 110527. 10.1016/j.jpsychores.2021.110527. 34082154 PMC9009535

[ejp70266-bib-0017] Gibbons, C. J. 2017. “Turning the Page on Pen‐and‐Paper Questionnaires: Combining Ecological Momentary Assessment and Computer Adaptive Testing to Transform Psychological Assessment in the 21st Century.” Frontiers in Psychology 7: 1933. 10.3389/fpsyg.2016.01933. 28154540 PMC5244429

[ejp70266-bib-0018] Gopinath, B. , J. Jagnoor , A. Kifley , et al. 2019. “Differential Predictors of Pain Severity Over 12 Months Following Noncatastrophic Injury Sustained in a Road Traffic Crash.” Journal of Pain 20, no. 6: 676–684. 10.1016/j.jpain.2018.11.011. 30529696

[ejp70266-bib-0019] Hu, L. T. , and P. M. Bentler . 1999. “Cutoff Criteria for Fit Indexes in Covariance Structure Analysis: Conventional Criteria Versus New Alternatives.” Structural Equation Modeling: A Multidisciplinary Journal 6, no. 1: 1–55. 10.1080/10705519909540118.

[ejp70266-bib-0020] Intille, S. , C. Haynes , D. Maniar , A. Ponnada , and J. Manjourides . 2016. “μEMA: Microinteraction‐Based Ecological Momentary Assessment (EMA) Using a Smartwatch.” In *Proceedings of the 2016 ACM International Joint Conference on Pervasive and Ubiquitous Computing*, 2016, 1124–1128. 10.1145/2971648.2971717. PMC6143290 30238088

[ejp70266-bib-0021] May, M. , D. U. Junghaenel , M. Ono , A. A. Stone , and S. Schneider . 2018. “Ecological Momentary Assessment Methodology in Chronic Pain Research: A Systematic Review.” Journal of Pain 19, no. 7: 699–716. 10.1016/j.jpain.2018.01.006. 29371113 PMC6026050

[ejp70266-bib-0022] Meyer, K. , H. Sprott , and A. F. Mannion . 2008. “Cross‐Cultural Adaptation, Reliability, and Validity of the German Version of the Pain Catastrophizing Scale.” Journal of Psychosomatic Research 64, no. 5: 469–478. 10.1016/j.jpsychores.2007.12.004. 18440399

[ejp70266-bib-0023] Morren, M. , S. van Dulmen , J. Ouwerkerk , and J. Bensing . 2009. “Compliance With Momentary Pain Measurement Using Electronic Diaries: A Systematic Review.” European Journal of Pain 13, no. 4: 354–365. 10.1016/j.ejpain.2008.05.010. 18603458

[ejp70266-bib-0024] Nahum‐Shani, I. , E. B. Hekler , and D. Spruijt‐Metz . 2015. “Building Health Behavior Models to Guide the Development of Just‐In‐Time Adaptive Interventions: A Pragmatic Framework.” Health Psychology 34: 1209–1219. 10.1037/hea0000306. PMC4732268 26651462

[ejp70266-bib-0025] Nahum‐Shani, I. , S. N. Smith , B. J. Spring , et al. 2018. “Just‐In‐Time Adaptive Interventions (JITAIs) in Mobile Health: Key Components and Design Principles for Ongoing Health Behavior Support.” Annals of Behavioral Medicine 52, no. 6: 446–462. 10.1007/s12160-016-9830-8. 27663578 PMC5364076

[ejp70266-bib-0026] Osman, A. , F. X. Barrios , P. M. Gutierrez , B. A. Kopper , T. Merrifield , and L. Grittmann . 2000. “The Pain Catastrophizing Scale: Further Psychometric Evaluation With Adult Samples.” Journal of Behavioral Medicine 23, no. 4: 351–365. 10.1023/a:1005548801037. 10984864

[ejp70266-bib-0027] Osman, A. , F. X. Barrios , B. A. Kopper , W. Hauptmann , J. Jones , and E. O'Neill . 1997. “Factor Structure, Reliability, and Validity of the Pain Catastrophizing Scale.” Journal of Behavioral Medicine 20, no. 6: 589–605. 10.1023/a:1025570508954. 9429990

[ejp70266-bib-0028] Patridge, E. F. , and T. P. Bardyn . 2018. “Research Electronic Data Capture (REDCap).” Journal of the Medical Library Association: JMLA 106: 142–144. 10.5195/jmla.2018.319.

[ejp70266-bib-0029] Reeve, B. B. , R. D. Hays , J. B. Bjorner , et al. 2007. “Psychometric Evaluation and Calibration of Health‐Related Quality of Life Item Banks: Plans for the Patient‐Reported Outcomes Measurement Information System (PROMIS).” Medical Care 45, no. 5: S22–S31. 10.1097/01.mlr.0000250483.85507.04. 17443115

[ejp70266-bib-0030] Rodebaugh, T. L. , R. B. Scullin , J. K. Langer , et al. 2016. “Unreliability as a Threat to Understanding Psychopathology: The Cautionary Tale of Attentional Bias.” Journal of Abnormal Psychology 125, no. 6: 840–851. 10.1037/abn0000184. 27322741 PMC4980228

[ejp70266-bib-0031] Rodriguez, A. , S. P. Reise , and M. G. Haviland . 2016. “Evaluating Bifactor Models: Calculating and Interpreting Statistical Indices.” Psychological Methods 21, no. 2: 137–150. 10.1037/met0000045. 26523435

[ejp70266-bib-0032] Rose, M. , J. Bjorner , F. Fischer , et al. 2012. “Computerized Adaptive Testing—Ready for Ambulatory Monitoring?” Psychosomatic Medicine 74, no. 4: 338–348. 10.1097/PSY.0b013e3182547392. 22582331

[ejp70266-bib-0033] Rosseel, Y. 2012. “Lavaan: An R Package for Structural Equation Modeling.” Journal of Statistical Software 48, no. 2: 1–36. 10.18637/jss.v048.i02.

[ejp70266-bib-0034] Samejima, F. 1997. “Graded Response Model.” In Handbook of Modern Item Response Theory, edited by W. J. van der Linden and R. K. Hambleton , 85–100. Springer‐Verlag. 10.1007/978-1-4757-2691-6_5.

[ejp70266-bib-0035] Schneider, S. , D. U. Junghaenel , J. M. Smyth , C. K. Fred Wen , and A. A. Stone . 2024. “Just‐In‐Time Adaptive Ecological Momentary Assessment (JITA‐EMA).” Behavior Research Methods 56, no. 2: 765–783. 10.3758/s13428-023-02083-8. 36840916 PMC10450096

[ejp70266-bib-0036] Shiffman, S. , A. A. Stone , and M. R. Hufford . 2008. “Ecological Momentary Assessment.” Annual Review of Clinical Psychology 4: 1–32. 10.1146/annurev.clinpsy.3.022806.091415. 18509902

[ejp70266-bib-0037] Sitthipornvorakul, E. , T. Klinsophon , R. Sihawong , and P. Janwantanakul . 2018. “The Effects of Walking Intervention in Patients With Chronic Low Back Pain: A Meta‐Analysis of Randomized Controlled Trials.” Musculoskeletal Science & Practice 34: 38–46. 10.1016/j.msksp.2017.12.003. 29257996

[ejp70266-bib-0038] Stone, A. A. , S. Schneider , and J. M. Smyth . 2023. “Evaluation of Pressing Issues in Ecological Momentary Assessment.” Annual Review of Clinical Psychology 19: 107–131. 10.1146/annurev-clinpsy-080921-083128. PMC12991416 36475718

[ejp70266-bib-0039] Sullivan, M. J. , and J. L. D'Eon . 1990. “Relation Between Catastrophizing and Depression in Chronic Pain Patients.” Journal of Abnormal Psychology 99, no. 3: 260–263. 10.1037//0021-843x.99.3.260. 2145334

[ejp70266-bib-0040] Sullivan, M. J. , B. Thorn , J. A. Haythornthwaite , et al. 2001. “Theoretical Perspectives on the Relation Between Catastrophizing and Pain.” Clinical Journal of Pain 17, no. 1: 52–64. 10.1097/00002508-200103000-00008. 11289089

[ejp70266-bib-0041] Sullivan, M. J. L. , S. R. Bishop , and J. Pivik . 1995. “The Pain Catastrophizing Scale: Development and Validation.” Psychological Assessment 7, no. 4: 524–532. 10.1037/1040-3590.7.4.524.

[ejp70266-bib-0042] Sullivan, M. J. L. , M. E. Lynch , and A. J. Clark . 2005. “Dimensions of Catastrophic Thinking Associated With Pain Experience and Disability in Patients With Neuropathic Pain Conditions.” Pain 113, no. 3: 310–315. 10.1016/j.pain.2004.11.003. 15661438

[ejp70266-bib-0043] Van Damme, S. , G. Crombez , P. Bijttebier , L. Goubert , and B. Van Houdenhove . 2002. “A Confirmatory Factor Analysis of the Pain Catastrophizing Scale: Invariant Factor Structure Across Clinical and Non‐Clinical Populations.” Pain 96, no. 3: 319–324. 10.1016/s0304-3959(01)00463-8. 11973004

[ejp70266-bib-0044] Ziadni, M. S. , J. A. Sturgeon , and B. D. Darnall . 2018. “The Relationship Between Negative Metacognitive Thoughts, Pain Catastrophizing and Adjustment to Chronic Pain.” European Journal of Pain 22, no. 4: 756–762. 10.1002/ejp.1160. 29214679 PMC5854507

