# Supplementary material for: Enhancing High‐Resolution Assessment in Pain Disorders: Development of an Adaptive Real‐Time Version of the Pain Catastrophizing Scale
Source: Eur J Pain. 2026 Apr 20;30:e70266. doi: 10.1002/ejp.70266 (PMC13094327; doi:10.1002/ejp.70266)
Supplement: Supplementary file 1 — Table S1: German changes and version of the momentary PCS. [file EJP-30-0-s001.docx]

**Table S1.**  German changes and version of the momentary PCS

In Bezug auf meine Schmerzen beschäftigen mich im Moment folgende Gedanken…

| **Dimension** | **Item Nr.** | **Content** | **trifft**  **überhaup**  **nicht zu** | **trifft**  **eher**  **nicht**  **zu** | **Teils-teils** | **trifft**  **eher**  **zu** | **trifft**  **~~Immer~~**  **sehr**  **zu** |
| --- | --- | --- | --- | --- | --- | --- | --- |
| Hilflosigkeit | 1 | Ich mache mir ~~ständig~~ Sorgen, ob die Schmerzen wohl jemals wieder aufhören werden. |  |  |  |  |  |
| Hilflosigkeit | 2 | Ich denke, ich kann nicht mehr. |  |  |  |  |  |
| Hilflosigkeit | 3 | Der Zustand ist schrecklich und ich denke, dass es nie mehr besser wird. |  |  |  |  |  |
| Hilflosigkeit | 4 | Der Zustand ist furchtbar und droht mich zu überwältigen. |  |  |  |  |  |
| Hilflosigkeit | 5 | Ich habe das Gefühl, ich halte es nicht mehr aus. |  |  |  |  |  |
| Verstärkung | 6 | Ich ~~bekomme~~ habe Angst, dass die Schmerzen noch stärker werden. |  |  |  |  |  |
| Verstärkung | 7 | Ich denke ~~ständig~~ an andere Situationen, in denen ich Schmerzen hatte. |  |  |  |  |  |
| Grübeln | 8 | Ich wünsche mir verzweifelt, dass die Schmerzen weggehen. |  |  |  |  |  |
| Grübeln | 9 | Ich kann nicht aufhören, an die Schmerzen zu denken. |  |  |  |  |  |
| Grübeln | 10 | Ich denke ~~ständig~~ daran, wie sehr es schmerzt. |  |  |  |  |  |
| Grübeln | 11 | Ich denke ~~ständig~~ daran, wie sehr ich mir ein Ende der Schmerzen herbeiwünsche. |  |  |  |  |  |
| Grübeln | 12 | Es gibt nichts, was ich tun kann, um die Schmerzen zu lindern. |  |  |  |  |  |
| Verstärkung | 13 | Ich mache mir Sorgen, dass die Schmerzen auf etwas Schlimmes hindeuten. |  |  |  |  |  |
