# Supplementary material for: Enhancing High‐Resolution Assessment in Pain Disorders: Development of an Adaptive Real‐Time Version of the Pain Catastrophizing Scale
Source: Eur J Pain. 2026 Apr 20;30:e70266. doi: 10.1002/ejp.70266 (PMC13094327; doi:10.1002/ejp.70266)
Supplement: Supplementary file 2 — Table S2: English changes and version of the momentary PCS. [file EJP-30-0-s002.docx]

**Table S2.**  English changes and version of the momentary PCS

~~When I have pain, the following thoughts come to my mind~~

With regard to my pain, the following thoughts are currently on my mind

| **Dimension** | **Item Nr** | **Content** | **not at all** | **to a slight degree** | **to a moderate degree** | **to a great degree** | **~~all the time~~**  **Very much** |
| --- | --- | --- | --- | --- | --- | --- | --- |
| Helplessness | 1 | I worry ~~all the time about~~ whether the pain will end. |  |  |  |  |  |
| Helplessness | 2 | I feel I can’t go on. |  |  |  |  |  |
| Helplessness | 3 | It’s terrible and I think it’s never going to get any better. |  |  |  |  |  |
| Helplessness | 4 | It’s awful and I feel that it overwhelms me. |  |  |  |  |  |
| Helplessness | 5 | I feel I can’t stand it anymore. |  |  |  |  |  |
| Magnification | 6 | I ~~become~~ am afraid that the pain will get worse |  |  |  |  |  |
| Magnification | 7 | I ~~keep~~ think~~ing~~ of other painful events. |  |  |  |  |  |
| Rumination | 8 | I anxiously want the pain to go away. |  |  |  |  |  |
| Rumination | 9 | I can’t seem to keep it out of my mind. |  |  |  |  |  |
| Rumination | 10 | I ~~keep~~ think~~ing~~ about how much it hurts |  |  |  |  |  |
| Rumination | 11 | I ~~keep~~ think~~ing~~ about how badly I want the pain to stop. |  |  |  |  |  |
| Rumination | 12 | There’s nothing I can do to reduce the intensity of the pain. |  |  |  |  |  |
| Magnification | 13 | I wonder whether something serious may happen |  |  |  |  |  |
